# Measurement error of the Mini-Mental State Examination among individuals with dementia that reside in nursing homes

**DOI:** 10.1007/s10433-020-00572-9

**Published:** 2020-06-03

**Authors:** Carl Hörnsten, Håkan Littbrand, Gustaf Boström, Erik Rosendahl, Lillemor Lundin-Olsson, Peter Nordström, Yngve Gustafson, Hugo Lövheim

**Affiliations:** 1 Department of Community Medicine and Rehabilitation, Geriatric Medicine, Umeå University, Umeå, Sweden; 2 Department of Community Medicine and Rehabilitation, Physiotherapy and Geriatric Medicine, Umeå University, Umeå, Sweden; 3 Department of Community Medicine and Rehabilitation, Physiotherapy, Umeå University, Umeå, Sweden

**Keywords:** Absolute reliability, Mini-Mental State Examination, Test–retest reliability, Intra-rater reliability, Dementia, Nursing homes

## Abstract

Few studies have investigated the measurement error of the Mini-Mental State Examination (MMSE) in the same unit of measurement, also known as absolute reliability. This measurement can help determine whether an observed score change for an individual is likely to represent true change. The aim of this study was to investigate the absolute reliability of the MMSE among individuals with dementia that reside in nursing homes. Among 88 participants, 19 (21.6%) were men, 35 (39.8%) had Alzheimer’s disease, 35 (39.8%) had vascular dementia, and the mean age was 84.0 years (range 65–98). The participants were tested and retested with the MMSE within 1–6 days. Both tests were administered by the same assessor at the same time of day. The mean MMSE score was 13.7 (range 0–28). The absolute difference between MMSE scores varied from 0 to 6 points, and the differences did not correlate with the corresponding score means (*p* = 0.874). The smallest detectable change (SDC) between two measurements was 4.00. The SDC was independent of depression, impaired vision and hearing, delirium within the last week, dementia type and age. However, the SDC was 5.56 among men and 3.50 among women (*p* = 0.003). In conclusion, for individuals with dementia that reside in nursing homes, it seems like their MMSE score needs to change by four or more points between two measurements in order for their score change to be reliably higher than the measurement error.

**Electronic supplementary material:**

The online version of this article (10.1007/s10433-020-00572-9) contains supplementary material, which is available to authorized users.

## Background

The psychometric properties of the Mini-Mental State Examination (MMSE) (Folstein et al. 1975) have been extensively investigated (Tombaugh and McIntyre 1992; Monroe and Carter 2012), yet few studies have investigated the measurement error of the MMSE. A quantification of the measurement error of an assessment presented in the same unit of measurement, also known as absolute reliability, can help determine whether an observed score change for an individual is likely to represent true change. The MMSE has been used in the clinical management of dementia to evaluate disease progression, although it may not be suitable for this task if its absolute reliability is unknown or unacceptable.

In a previous multicenter study that was conducted in the USA for individuals with Alzheimer’s disease, 95% of the MMSE retest scores were within six points of their original scores over a 1-month period. Based on data presented in the study, the smallest detectable change (SDC) of the MMSE can be calculated to be 5.5 points (Clark et al. 1999). While many participants were recruited for this study, it excluded a broad range of individuals with comorbid conditions. In addition, the mean age of the participants was 71 years and a majority of the participants were community-dwelling (Clark et al. 1999; Fillenbaum et al. 2008). To our knowledge, the absolute reliability of the MMSE has not been investigated among individuals with dementia that reside in nursing homes. This information would be useful to accurately describe progression of cognitive impairments in an individual patient and to evaluate the individual benefits of health care interventions, including medication, social stimulation and physical exercise.

The primary aim of this study was to investigate the absolute test–retest reliability of the MMSE among individuals with dementia that live in nursing homes. The secondary aim was to investigate whether demographic factors, depression, delirium, dementia type or impairments of vision or hearing affect the absolute reliability of the MMSE, and to investigate the relative test–retest reliability of the MMSE.

## Methods

### Setting

The Umeå Dementia and Exercise (UMDEX) study was a randomized controlled trial designed to investigate the effects of physical exercise versus scheduled social activities among nursing home residents with dementia that reside in the city Umeå in northern Sweden (Toots et al. 2016). Screening and recruitment of suitable candidates with dementia started in August 2011, and the intervention period extended from October 2011 to January 2012. Absolute reliability of the MMSE was assessed for the present study in May 2012, 3 months after the intervention was completed. Ethical approval was obtained from the Regional Ethical Review Board in Umeå (2011-205-31M).

### Participants

People with dementia, aged 65 years or older, who were dependent in activities of daily living according to the Katz index (Katz et al. 1963), who were able to stand up from a chair with armrests with assistance of no more than one person, who were able to hear and understand spoken Swedish sufficiently to participate in assessments and who achieved MMSE scores of ten or higher during their initial cognitive screening, were invited to participate in the exercise trial. Of the 186 individuals who participated in the trial, 27 had passed away as of May 2012, 55 could not be tested and retested within 1 week due to a limited number of testers, one was acutely ill, one was in palliative care, eight did not want to participate, and six were excluded for other reasons. Therefore, the final sample in the present study included 88 individuals.

### Procedure

Four physiotherapists and two physicians, all trained and experienced, performed the primary data collection. For this, the MMSE was conducted and then repeated within 1 week by the same tester (range 1–6 days; mean, 2.3 ± 1.8 days; median, 1 day) at the same time of day on both occasions (± 2 h). Prior to each testing session, a staff member from the corresponding nursing home was asked whether the participant was acutely ill or whether any other temporary occurrence (e.g., lack of sleep or a fall) could have affected the participant’s typical level of functioning. If the level of functioning was compromised, the testing session was postponed. During the second testing session, a staff member from the nursing home responded to questions from the confusion subscale of the Organic Brain Syndrome scale (Jensen et al. 1993) to measure the prevalence of delirium within 1 week of retesting. The testers did not have access to the form that was completed from the first test session when they started the second test session.

### Assessment scales

The MMSE (Folstein et al. 1975) is an assessment of cognitive ability which includes test items that refer to: “orientation to time,” “orientation to place,” “registration,” “attention and calculation,” “recall,” “language,” “repetition,” “three-stage command,” “reading and obey,” “writing complete sentence” and “copying figure.” Scoring for the MMSE ranges from 0 to 30, and scores ≤ 23 are considered to indicate cognitive impairment. A Swedish version of the original MMSE, which was translated by the Swedish Association for Cognitive Disorders, was used. This version of the MMSE has been used extensively in Sweden both in research and in clinical practice. Regarding the item, “attention and calculation,” the participants were asked to perform the test serial sevens. However, those who outright refused to answer were asked to spell a word backwards instead.

The Organic Brain Syndrome scale (Jensen et al. 1993) is a test for organic brain disorders in older populations. Here, only the confusion subscale was used.

The 15-item version of the Geriatric Depression Scale (GDS) (Sheikh and Yesavage 1986) is a test for symptoms of depression in older individuals. A score of ≥ 5 is considered to indicate depression.

### Medical diagnoses and definitions

A team of physicians, including a geriatric medicine specialist, established dementia diagnoses according to DSM-IV-TR criteria using medical records, MMSE scores, assessment of temporary states of confusion, and information regarding visual and hearing impairment. Classification of dementia type was based on medical records, and in most cases, the records included brain imaging, anamnesis of memory impairment, a history of other diseases and past MMSE scores. The latter were compared with MMSE scores obtained for this study. Dementia diagnoses were decided during the screening phase of the selection process.

Delirium was considered present if any item on the confusion subscale of the OBS, other than the item regarding tiredness, received a nonnegative answer. All participants were possible to assess with the OBS.

Depression was considered present if the participant achieved a score of ≥ 5 on the GDS, assessed in May 2012. Two participants were not possible to assess with the GDS.

All other medical diagnoses were based on review of medical records up until the day when MMSE was first tested for the present study for each participant.

Visual impairment was considered present if the participant was unable to read capitalized text of the size 5 mm with or without glasses. Hearing impairment was considered present if the participant was unable to hear normal loudness of speech from a distance of 1 meter with or without hearing aids.

### Statistics

R 3.0.1 was used to perform the statistical analyses and to generate the figures for this study. Categorical variables were compared with the Chi-squared test or Fisher’s exact test, and numerical variables were compared with the Mann–Whitney *U* test. A paired test was used to compare the mean MMSE scores calculated from the original tests and the retests.

Relative reliability was evaluated with intra-class correlation coefficients (ICCs) with both subjects and raters considered to represent random effects [ICC(2,1)]. Reliability of the individual MMSE items was investigated with weighted kappa coefficients. Absolute reliability was evaluated according to a previously published multi-step process (Bland and Altman 1996). A visual examination of the plots generated from the score differences between the test and retest data against their means was performed to examine the assumption that the measurement error does not depend on the size of the measurements taken. This assumption was also tested by performing the Kendall correlation test of the absolute test–retest differences against their means. Within-subject standard deviation (*s*_w_) was calculated by dividing the standard deviation (SD) of the score difference between each test and retest by the square root of two. The SDC between two measurements was calculated by multiplying *s*_w_ by the square root of two times 1.96 (e.g., 2.77). There were no observed score differences between the test and retest scores that represented extreme outliers (e.g., > 3 * interquartile range). Variances in MMSE score differences, and consequently SDC differences, were compared between subgroups using Levene’s test.

## Results

Baseline characteristics of the cohort examined are listed in Table 1. Of the 88 participants, 35 (39.8%) had Alzheimer’s disease, 35 (39.8%) had vascular dementia, 7 (8.0%) had mixed dementia (Alzheimer’s disease and vascular dementia), 4 (4.5%) had other types of mixed dementia, 3 (3.4%) had alcohol-related dementia, 1 had frontotemporal dementia, and 3 had unspecified dementia. The mean age of the participants was 84.0 ± 7.4 y (range 65–98), and 19 participants (21.6%) were men. Diabetes was more common among male than among female participants (*p* < 0.001) (Table 1).

**Table 1:** Characteristics of the participants

	Total *n* = 88	Men *n* = 19	Women *n* = 69	*p* value
Age (years) ± SD	84.0 ± 7.4	81.7 ± 7.6	84.6 ± 7.2	0.131
Days between tests ± SD	2.3 ± 1.8	2.3 ± 1.7	2.3 ± 1.9	0.940
Impaired vision, *n* (%)	13 (14.8)	2 (10.5)	11 (15.9)	0.726^FE^
Impaired hearing, *n* (%)	22 (25.0)	5 (26.3)	17 (24.6)	1.000^FE^
Delirium (1 week), *n* (%)	50 (56.8)	12 (63.2)	38 (55.1)	0.712
Alzheimer’s disease, *n* (%)	35 (39.8)	7 (36.8)	28 (40.6)	0.976
Vascular dementia, *n* (%)	35 (39.8)	7 (36.8)	28 (40.6)	0.976
Other dementia, *n* (%)	18 (20.4)	5 (26.4)	13 (18.8)	0.525^FE^
Depression (GDS ≥ 5), *n* (%)	22 (25.6)	5 (26.3)	17 (25.4)	1.000^FE^
Stroke, *n* (%)	28 (31.8)	8 (42.1)	20 (29.0)	0.418
Diabetes, *n* (%)	15 (17.0)	9 (47.4)	6 (8.7)	< 0.001^FE^
Hypertension, *n* (%)	53 (60.2)	11 (57.9)	42 (60.9)	1.000
Myocardial infarction, *n* (%)	22 (25.0)	7 (36.8)	15 (21.7)	0.232^FE^
Heart failure, *n* (%)	25 (28.4)	7 (36.8)	18 (26.1)	0.527
Malignancy, *n* (%)	18 (20.5)	5 (26.3)	13 (18.8)	0.525^FE^

*FE* Fisher’s exact test was applied in place of the Chi-squared test if the expected count was < 5 for any combination of variables. *GDS* Geriatric Depression Scale 15-item version

**Fig. 1:**
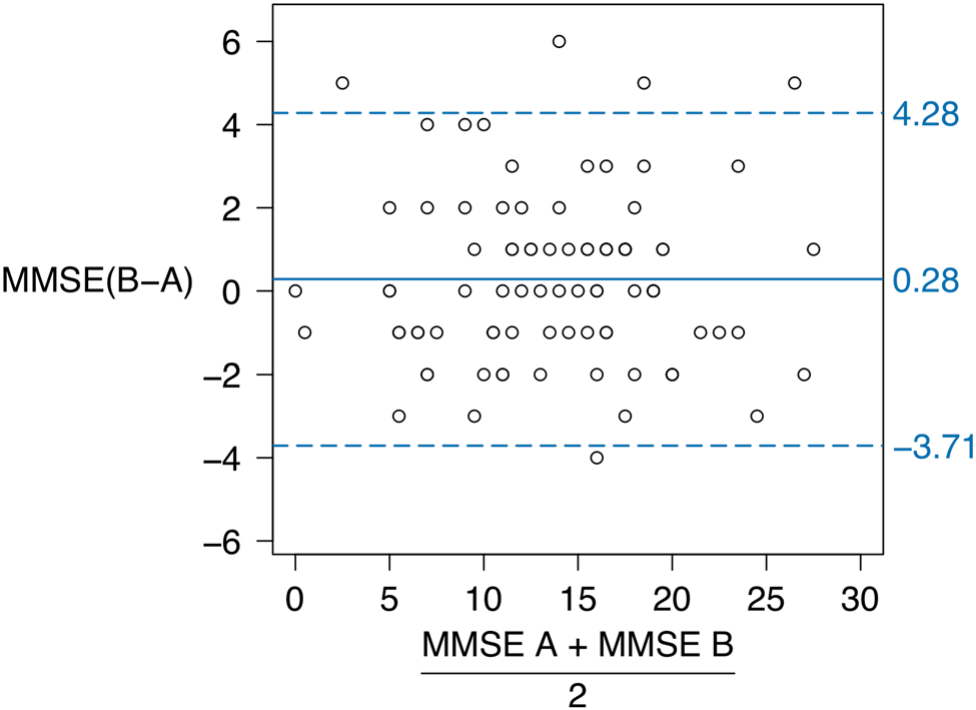
Differences in MMSE scores between the initial tests (A) and the retests (B) plotted versus score means. The solid line represents the mean difference. The dashed lines represent the addition (upper) and subtraction (lower) of the smallest detectable from the mean difference

The mean MMSE score at the initial test was 13.74 ± 5.92 (range 0–28) and it was 14.02 ± 5.93 (range 0–29) at the retest (*p* = 0.395). The absolute differences between the MMSE scores ranged from 0 to 6 points. When the differences between the MMSE scores were plotted against their means, no signs of heteroscedasticity were observed visually (Fig. 1) or were identified when tested (*p* = 0.874). The mean MMSE score difference between the tests was 0.28 ± 2.04, and the within-subject standard deviation was 1.44. Thus, the SDC between the two measurements was 3.998 (Table 2).

**Table 2:** Absolute reliability of the MMSE

	Total *n* = 88	Men *n* = 19	Women *n* = 69
MMSE test score, mean ± SD	13.74 ± 5.92	13.74 ± 6.21	13.74 ± 5.89
MMSE retest score, mean ± SD	14.02 ± 5.93	14.21 ± 6.01	13.97 ± 5.95
MMSE difference, mean ± SD	0.28 ± 2.04	0.47 ± 2.84*	0.23 ± 1.78*
Within-subject standard deviation, *s*_w_	1.44	2.01	1.26
Smallest detectable change	4.00	5.56	3.50

*Levene’s test, *p* = 0.003

Additional comparisons between subgroups showed that the SDC for the MMSE scores was independent of depression (*p* = 0.745), impaired vision (*p* = 0.473), impaired hearing (*p* = 0.370), delirium within 1 week of retesting (*p* = 0.762), Alzheimer’s disease compared with vascular dementia (*n* = 70; *p* = 0.109) or among the ≥ 85-year-olds compared with < 85-year-olds (*p* = 0.637). The SDC did not differ when comparing the six testers (*p* = 0.924) or median or longer follow-up intervals compared to shorter than median follow-up intervals (*p* = 0.821). However, the SDC was 5.56 among the male participants and 3.50 among the female participants (*p* = 0.003) (Table 2).

The relative reliability of the MMSE according to the calculated ICCs was 0.94 overall, 0.89 among men and 0.95 among women. The reliability of the specific MMSE items based on weighted kappa statistics was ≥ 0.8 for “orientation to place,” “attention and calculation” and “recall,” between 0.6 and 0.8 for “orientation to time,” “language,” “repetition,” “reading and obey” and “writing complete sentence,” and < 0.6 for “registration,” “three-stage command” and “copying figure” (Table 3). Among the items with the lowest reliability scores, “registration” and “three-stage command” exhibited relatively large item-specific mean differences, while “copying figure” was not similarly affected (Table 3).

**Table 3:** Absolute score differences and relative reliability of individual items of the MMSE

Items	Range	Test score Mean ± SD	Retest score Mean ± SD	Difference Mean ± SD	Weighted kappa coefficient (CI)
1. Orientation to time	0–5	1.28 ± 1.35	1.34 ± 1.37	0.06 ± 1.09	0.68 (0.54–0.82)
2. Orientation to place	0–5	2.82 ± 1.43	2.82 ± 1.50	0.00 ± 0.92	0.80 (0.71–0.89)
3. Registration	0–3	2.50 ± 0.97	2.73 ± 0.64	0.23 ± 0.74	0.57 (0.36–0.79)
4. Attention and calculation	0–5	0.94 ± 1.33	0.91 ± 1.35	− 0.03 ± 0.60	0.89 (0.82–0.96)
5. Recall	0–3	0.61 ± 0.93	0.64 ± 1.05	0.02 ± 0.57	0.84 (0.75–0.93)
6. Language	0–2	1.84 ± 0.48	1.83 ± 0.48	− 0.01 ± 0.39	0.68 (0.42–0.93)
7. Repetition	0–1	0.36 ± 0.48	0.33 ± 0.47	− 0.03 ± 0.41	0.62 (0.45–0.80)
8. Three-stage command	0–3	1.81 ± 1.03	1.92 ± 1.00	0.11 ± 0.92	0.59 (0.44–0.73)
9. Reading and obey	0–1	0.76 ± 0.43	0.76 ± 0.43	0.00 ± 0.37	0.62 (0.43–0.82)
10. Writing complete sentence	0–1	0.57 ± 0.50	0.55 ± 0.50	− 0.02 ± 0.43	0.63 (0.47–0.79)
11. Copying figure	0–1	0.24 ± 0.43	0.20 ± 0.41	− 0.03 ± 0.47	0.38 (0.15–0.60)

*CI* confidence interval

## Discussion

The results of the present study suggest that for individuals with dementia residing in nursing homes, their MMSE scores need to change by ≥ 4 points between two measurements for their score change to be considered reliably larger than the measurement error. Moreover, depression, delirium within the previous week, age, dementia type and hearing or visual impairments did not seem to affect measurement error, although the measurement error was larger for men than for women in the present cohort.

The SDC of the MMSE among the individuals with dementia that were examined in the present study was lower when compared with a previous multicenter study (Clark et al. 1999), which found a SDC of 5.5 over a 1-month period. This difference may indicate that the MMSE is better at detecting individual score changes among individuals with dementia that reside in nursing homes than among individuals that reside in community settings. This is somewhat surprising considering that many individuals that reside in nursing homes suffer from multiple morbidities and they have a lower reserve capacity, thereby increasing their sensitivity to both internal and external interferences (Campbell and Buchner 1997). Correspondingly, larger variations in functional ability would be predicted compared to individuals that live in community settings. It may also be that the difference is due to the shorter interval between the tests and retests that were conducted, the efforts to disregard the effects of temporary disease processes, and/or the matching of the times of the tests and retests in the present study that could have resulted in a more accurate estimate of absolute reliability. To our knowledge, there are no additional studies that are directly comparable to the present study. However, based on two separate tests from a study of cognitively intact community dwellers, the SDC of the MMSE over a 3-month period can be calculated as 4.55 points and 4.72 points (Tombaugh 2005). Additionally, the aforementioned multicenter study found a SDC of 2.5 for their dementia-free control group (Clark et al. 1999).

The MMSE appears to be useful for assessing cognitive change among individuals with dementia residing in nursing homes, also in individuals with visual or hearing impairments, depression or delirium, and among the very old. However, the measurement error for the MMSE in the present study was larger for men than for women. The only notable sex difference in characteristics was that more men had diabetes compared with the women, although this characteristic is unlikely to have caused the difference in measurement error. It should also be noted that the sample size of men was much smaller than that for the women, and this could have contributed to the results obtained. From a clinical perspective, it may be appropriate to make a distinction between MMSE score changes that are indistinguishable from the measurement error and changes that are likely to be true when describing nursing home patients with dementia. It should be made clear, however, that group level MMSE changes, which are widely studied in science, can be meaningful even if they are smaller than the smallest detectable change.

The high relative reliability of the MMSE according to the ICCs calculated in the present study is in accordance with previously published results (Tombaugh and McIntyre 1992). Most of the individual MMSE items had an acceptable reliability according to weighted kappa statistics, except for “copying picture.” The lower reliability of the latter item may be related to the relatively low success rate among the participants and the dichotomized evaluation of success, which could have led to notable random differences between the two tests.

The MMSE items “registration” and “three-stage command” showed signs of learning effects. However, the mean difference in MMSE scores for the entire assessment was only 0.28, indicating that learning effects were negligible overall.

## Limitations

It should be noted that there are several versions of the MMSE that differ in regard to which questions are asked and how they are asked. The present study used a Swedish translation of the MMSE that has been widely used in Sweden for both clinical and research applications. Therefore, the present results may not be generalizable to versions of the MMSE that have been heavily edited compared with the original version.

The recruitment of participants from within an intervention study may also have influenced the generalizability of the present results. Individuals with severe cognitive impairment (MMSE < 10) as well as those unable to stand up from a chair with armrests with assistance of no more than one person were possibly underrepresented in the present study, given the exclusion criteria from the intervention study. On the other hand, many individuals experienced cognitive decline after the intervention was started, and thus, there were many individuals affected by severe cognitive decline when absolute reliability of the MMSE was assessed. This situation is apparent from the data presented in Fig. 1. There were relatively fewer individuals on the extreme low and high ends of possible MMSE scores, which may have affected the generalizability of our conclusions regarding individuals with such scores.

Additionally, the participants showed heterogeneity regarding comorbidity from other diseases and disabilities. It is to be expected that individuals with dementia that reside in nursing homes suffer from significant comorbidity, but patterns of comorbidity may differ depending on how nursing home care is organized in different countries, which may affect the generalizability of our results.

The testing procedure used in the present study was standardized. Experienced testers conducted assessments according to a detailed protocol, with the two MMSE tests for each participant performed at the same time of day (± 2 h), and the tests being postponed if any temporary occurrence could have affected the participant’s typical level of functioning as reported by nursing home staff, with no more than 7 days between the tests. Considering the age and multiple illnesses of the participants in the present cohort, standardization procedures were used to maintain consistency. Despite our efforts, there may have been a temporary occurrence (e.g., lack of sleep or a fall) that could have affected the participant’s typical level of functioning between the tests and that were not obvious to nursing home staff. Deviations between measurements may be larger with non-standardized testing procedures, including the use of different testers. The absolute test–retest reliability did not differ between the six testers in the present study; however, inter-rater reliability was not investigated, which would have required multiple assessments of participants by different testers.

Comparisons among subgroups of the participants should be interpreted with some caution considering the low number of participants, especially in some investigated subgroups. A larger sample size, or the use of different inclusion criteria to achieve more uniform group sizes, may have provided different results.

## Conclusions

For individuals with dementia that reside in nursing homes, it seems like their MMSE score need to change by four or more points between two measurements in order for their scores to reliably indicate a change distinct from measurement error. In addition, depression, delirium within the previous week, dementia type, age and hearing or visual impairments did not affect measurement error among the MMSE scores examined. A possible sex-based difference in the measurement error of the MMSE should be investigated in future studies.

## Electronic supplementary material

Below is the link to the electronic supplementary material.

Supplementary material 1 (CSV 8 kb)
